# Identification of proteins that bind extracellular microRNAs secreted by the parasitic nematode *Trichinella spiralis*

**DOI:** 10.1242/bio.060096

**Published:** 2023-11-09

**Authors:** Alice Brown, Murray E. Selkirk, Peter Sarkies

**Affiliations:** ^1^ MRC London Institute of Medical Sciences, Du Cane Road, London, W12 0NN, UK; ^2^ Department of Life Sciences, Imperial College London, SW7 2AZ, UK; ^3^ Department of Biochemistry, University of Oxford, South Parks Road, Oxford, OX1 3QU, UK

**Keywords:** RNA binding protein, Extracellular, Helminth, MiRNA, Parasite

## Abstract

Small non-coding RNAs such as microRNAs (miRNAs) are conserved across eukaryotes and play key roles in regulating gene expression. In many organisms, miRNAs are also secreted from cells, often encased within vesicles such as exosomes, and sometimes extravesicular. The mechanisms of miRNA secretion, how they are stabilised outside of cells and their functional importance are poorly understood. Recently, we characterised the parasitic nematode *Trichinella spiralis* as a model to study miRNA secretion. *T. spiralis* muscle-stage larvae (MSL) secrete abundant miRNAs which are largely extravesicular. Here, we investigated how *T. spiralis* miRNAs might remain stable outside of cells. Using proteomics, we identified two RNA binding proteins secreted by *T. spiralis* larvae and characterised their RNA binding properties. One, a homologue of the known RNA binding protein KSRP, binds miRNA in a selective and sequence-specific fashion. Another protein, which is likely a novel RNA binding protein, binds to miRNA without exhibiting sequence specificity. Our results suggest a possible mechanism for miRNA secretion by *T. spiralis* and may have relevance for understanding the biology of extracellular miRNA more widely.

## Teaser

**Summary:** The parasitic nematode Trichinella spiralis secretes microRNAs that are not contained within extracellular vesicles. Here we discover and characterize two proteins that may be involved in stabilising these microRNAs.

## INTRODUCTION

Small (16-36-nt) non-coding RNAs (sRNAs) are key regulators of gene expression conserved across eukaryotes. Different pathways generate functionally distinct classes of sRNAs (Ketting and Cochella, 2021) but in general sRNAs associate with Argonaute proteins, which catalyse efficient recognition of target sites within RNAs through sense-antisense base pairing (Swarts et al., 2014). This then usually results in downregulation of the target RNA. microRNAs are one of the most abundant classes of sRNAs (Bartel, 2018). miRNAs are conserved in all animals and their activity has been demonstrated to be essential for successful development in several organisms (Kraus et al., 2017). miRNAs also play important roles in maintaining gene expression in differentiated cells and transcriptional responses to external stimuli. Target recognition by miRNA/Argonaute complexes leads to gene expression changes by inducing mRNA degradation and through disrupting translation (Dexheimer and Cochella, 2020).

Intracellular functions and mechanisms of miRNAs have been extensively characterised across animal species (Dexheimer and Cochella, 2020). However, much more mysterious is whether miRNAs might have functions outside cells. Interest in this area began with the unambiguous demonstration that sRNAs, including miRNAs, are transported between tissues in plants (Voinnet and Baulcombe, 1997) and nematodes (Winston et al., 2002). In animals, secretion of extracellular miRNAs has been observed from a wide variety of different tissues and cultured cells, and stable extracellular miRNAs have been identified in many different extracellular fluids such as saliva, breast milk and urine (Liu et al., 2019). There has been much speculation that these extracellular RNAs could be important in cell-to-cell communication. However, evidence for cell-to-cell transfer of miRNAs in animals is limited and there are many doubts over whether extracellular miRNAs can be delivered in sufficient quantities to exert changes in gene expression (Gruner and McManus, 2021). Importantly, there is also limited evidence that extracellular miRNAs are bound to Argonaute proteins, thus exactly how they would integrate into gene expression control mechanisms in recipient cells is unclear.

Lack of understanding about the functions of extracellular miRNAs is accompanied by considerable uncertainty over the mechanism whereby intracellular miRNAs are targeted for secretion and stabilised when in the extracellular environment (Gruner and McManus, 2021). The dominant theory has been that miRNAs are enclosed within exosomes which protects them from extracellular nuclease activity (Groot and Lee, 2020). Some intracellular sorting proteins have been implicated in selecting miRNAs for export via this pathway (Groot and Lee, 2020). Although this is the most straightforward way to explain how stable miRNAs can exist outside of cells in the absence of Argonaute proteins, it is notable that up to 50% of mammalian extracellular miRNAs are not enclosed in vesicles (Turchinovich et al., 2011, 2016), so there may be other mechanisms involved. How these miRNAs remain stable is poorly understood (Gruner and McManus, 2021).

Parasitic nematodes have emerged as an interesting model to study the mechanism and function of extracellular RNAs (Buck et al., 2014; Tritten and Geary, 2018). Several species of parasitic nematodes secrete sRNAs, including abundant miRNAs. Similarly to mammals, a substantial fraction of secreted miRNAs are enclosed within vesicles (Coakley et al., 2015). Some evidence exists that miRNAs secreted by parasitic nematodes in vesicles could be taken up by host cells and potentially contribute to gene regulation (Coakley et al., 2017). Interestingly, an Argonaute protein has been shown to be secreted by the parasitic nematode *Heligmosomoides polygyrus* (Chow et al., 2019). However, this Argonaute protein binds a different class of small non-coding RNAs known as 22G-RNAs (Chow et al., 2019) so is unlikely to be involved in stabilisation or delivery of miRNAs.

Recently we developed the parasitic nematode *Trichinella spiralis* as a model system to study extracellular small non-coding RNAs. *T. spiralis* is unusual because its life cycle comprises both intracellular and extracellular parasitic phases. Adults mate and produce offspring as extracellular parasites in the gut, but offspring migrate to the muscle cells of the host where they encyst as an intracellular parasite, known as muscle stage larvae (MSL), the L1 stage. MSL remain in this state until the animal is predated on, whereby they are released in the digestive tract and develop into adults to complete the life cycle (Despommier, 1998). Infection by *T. spiralis* MSL leads to a number of changes in muscle cells, most notably cell cycle re-entry and extensive remodelling (Jasmer, 1993). These processes may involve direct manipulation of gene expression by factors secreted by *T. spiralis* MSL (Wu et al., 2013), which include abundant small non-coding RNAs (Taylor et al., 2020) Interestingly, *T. spiralis* MSL secrete miRNAs that are almost exclusively not contained within vesicles, while adult *T. spiralis* secrete predominantly vesicular miRNAs (Taylor et al., 2020).

In this work, we investigate the mechanism of secretion of extravesicular miRNAs by *T. spiralis*. In particular, we focus on the question of how secreted miRNAs are stabilised. Using proteomics we identified several secreted RNA binding proteins, and in this manuscript focussed further on two examples that we explored in more detail. One of these proteins is from a protein family never previously implicated in nucleic acid interactions. We show that these proteins bind miRNAs both *in vitro* and in *T. spiralis* MSL secretomes. One protein binds non-selectively to miRNAs and the other binds only to a subset of miRNA. Together our work provides new insights into how extracellular miRNAs are stabilised in parasitic nematodes and may have implications for understanding the mechanisms of miRNA secretion in these organisms.

## RESULTS

### *T. spiralis* secretome contains RNA binding proteins

**Fig. 1.**
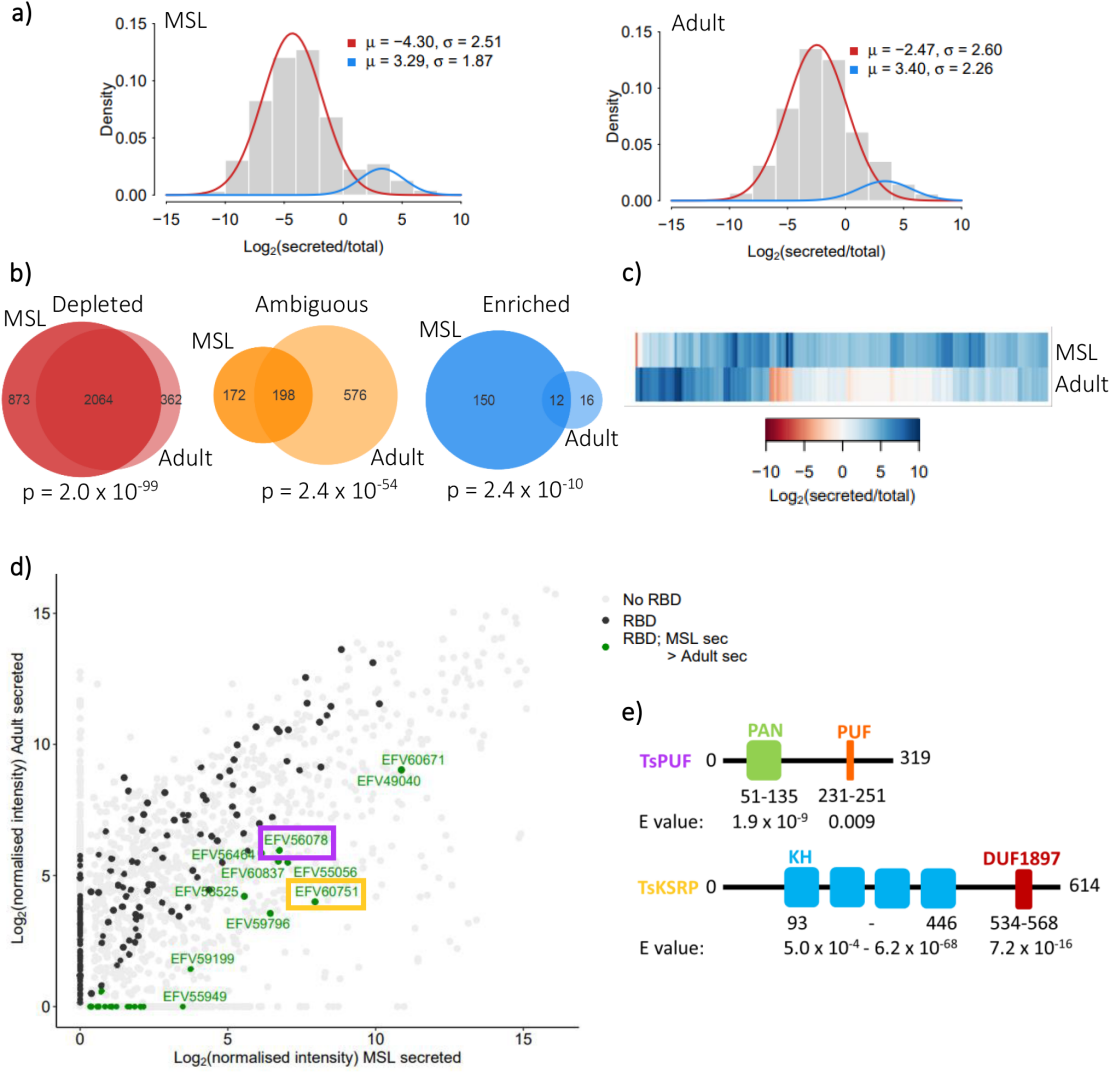
Comparison of *Trichinella spiralis* muscle-stage larvae (MSL) versus adult secretomes and identification of MSL abundantly secreted RNA-binding proteins. (A) Bimodal distribution of protein abundance in secreted material relative to in total worm in MSL and adults separately. Significant (*P*<0.05) posterior probabilities from an expectation maximisation algorithm were used to define proteins as either enriched (blue) or depleted (red) in the secreted material. Proteins with no significant posterior probability were defined as ambiguous. (B) Venn diagrams comparing numbers of proteins enriched, depleted or ambiguous in secreted material in MSL versus adults. *P* values obtained from Fisher's exact test of independence. (C) Heatmap comparing levels of enrichment of all 178 enriched secreted proteins in MSL versus adults. (D) Abundance, in MSL versus adults, of all proteins secreted by *T. spiralis.* Grey; no known RNA-binding domains (RBDs). Black; at least one RBD. Green; at least one RBD and more abundant in secreted material of MSL than that of adults (protein IDs labelled). Abundance of TsPUF (purple) and TsKSRP (orange) are highlighted. (E) Domain structure of the TsPUF and TsKSRP annotated by hmmscan. PUF, pumilio-*fem*-3 binding factor; KH, K homology domain; DUF1897, domain of unknown function 1897.

We previously showed that *T. spiralis* larvae secrete abundant miRNAs that are not enclosed in vesicles, leading to the question of how these miRNAs might be protected from nuclease activity (Taylor et al., 2020). We speculated that RNA binding proteins might be secreted alongside miRNAs and that these proteins might bind and stabilise miRNAs. We performed proteomics from secreted material from both adult and muscle stage larval (MSL) *T. spiralis* (Table S1). We identified subsets of proteins that were enriched in secreted material relative to whole-worm extracts (Fig. 1A). Extracting material from *T. spiralis* secretomes for proteomics is challenging, so we were only able to perform two replicate samples for each, so enrichment values are qualitative and not supported by *P*-values. However, we evaluated consistency between replicates for each protein and ensured that proteins selected for further analysis (see below) were amongst those with high consistency between replicates (Fig. S1). Although there was a significant overlap between proteins enriched in adult and MSL secretomes, some proteins were specifically enriched in the MSL secretome (Fig. 1B,C), indicating that they may be involved in stabilising extracellular miRNAs. We searched all proteins that were present in the secreted material from a manually curated list of RNA binding domains (Table S2). Several candidate proteins were identified which were enriched in MSL secreted material compared to adult secreted material (Fig. 1D; Table S3). We selected two of these proteins for further characterisation, on the basis of further bioinformatic characterisation which we describe below. One, which we refer to as TsPUF, had a region with weak similarity to the Pumilio homology (Puf) domain (Fig. 1E) (Goldstrohm et al., 2018). The other, which we refer to as TsKSRP, contained several matches to the KH domain present in many RNA binding proteins (Valverde et al., 2008).

### Bioinformatic characterisation of TsPUF and TsKSRP

**Fig. 2.**
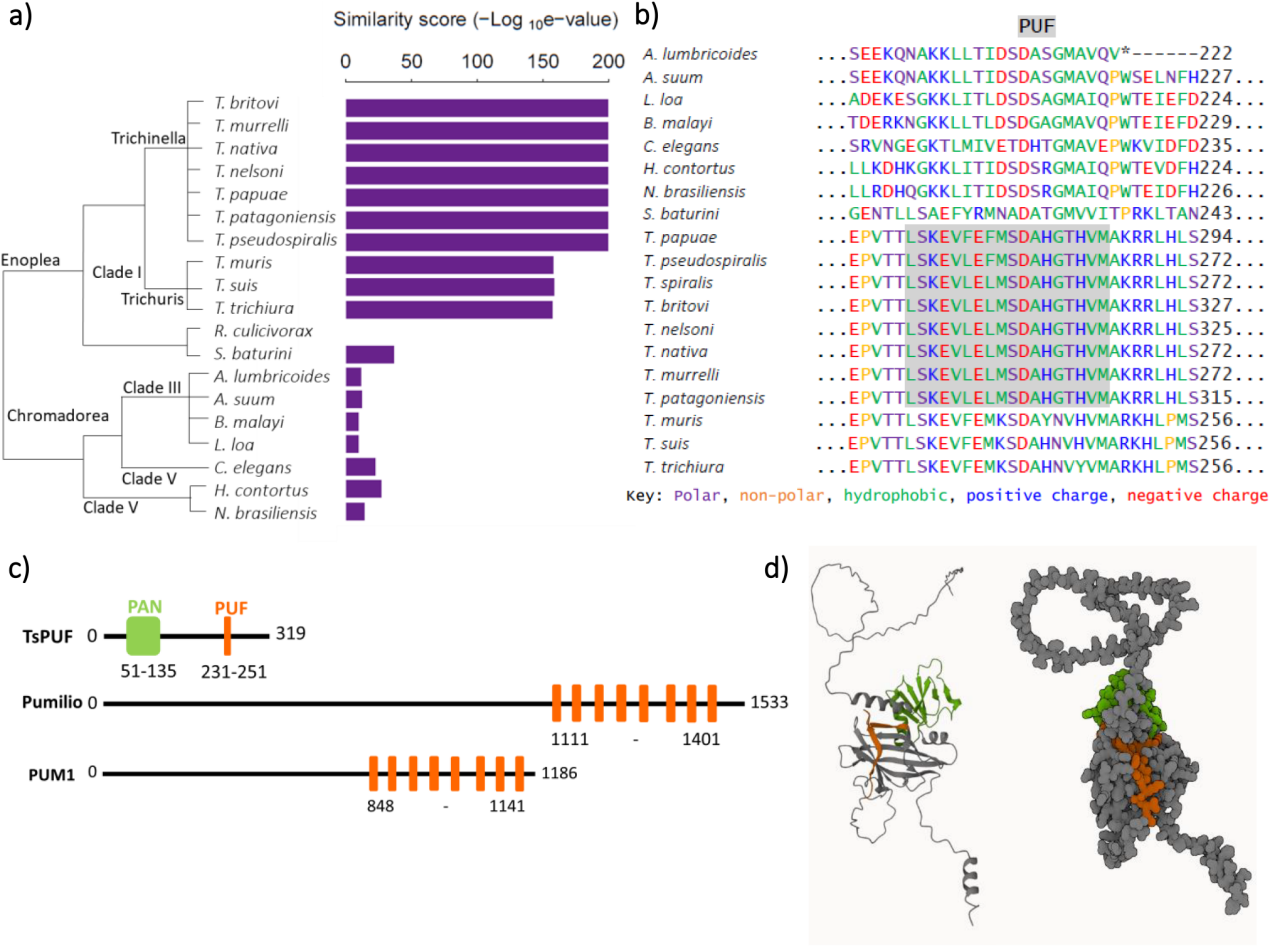
**Characterisation of TsPUF protein and structure.** (A) Similarity between TsPUF and its homologues in 19 nematode species; *Trichinella britovi, murrelli, nativa, nelsoni, papuae, patagoniensis* and *pseudospiralis, Trichuris muris, suis* and *trichiura, Romanomermis culicivorax, Soboliphyme baturini, Asacaris lumbricoides* and *suum, Brugia malayi, Loa loa, Caenorhabditis elegans, Haemonchus contortus* and *Nippostrongylus brasiliensis*. Homologues identified by performing a reciprocal best blast hit search. Similarity score=inverse log_10_ of the e value from the reciprocal best blast hit. (B) Alignment of the PUF region in TsPUF against the TsPUF nematode homologues. Multiple sequence alignment performed using Clustal Omega. Hmmscan used to identify Pfam domains. Amino acids are coloured according to their properties and the position of the PUF domain is highlighted. (C) Comparison of TsPUF protein domain structure versus that of two canonical PUF proteins; *Drosophila melanogaster* pumilio and *Homo sapiens* PUM1. (D) Alphafold prediction of TsPUF (A0A0V1BXK5_TRISP) structure. The PUF domain is coloured in orange and the PAN domain in green.

We characterised homologues of TsPUF across nematodes (Table S4), showing that TsPUF is widely conserved but that the region identified as the PUF domain was only evident within the *Trichinella* genus and a similar region within the *Trichuris* genus (Fig. 2A,B). *Trichinella pseudospiralis*, which is related to *T. spiralis* but unlike *T. spiralis* does not form capsules, also encodes a homologue of TsPUF with a similar PUF-like region and a signal peptide (Fig. 2B). The N terminus of TsPUF contained a canonical signal peptide, followed by a Panhandle (PAN) domain (Fig. 2C). The PAN domain is often found in extracellular proteins where it mediates protein–protein interactions (Tordai et al., 1999). These features suggested that TsPUF is most likely targeted to the extracellular environment through the canonical secretory pathway. Only one copy of the PUF domain was present (Fig. 2C), in contrast to known Pumilio homology domain proteins where several tandem PUF domains are found with each domain responsible for contacting one nucleic acid on the RNA target (Wang et al., 2001; Edwards et al., 2001). The PUF region in TsPUF is short and not widely conserved so is likely to have convergently evolved similarity to the PUF repeat found in Pumilio family members. However, the presence of a sequence with similarity to the PUF repeat suggested that it might nevertheless have RNA binding properties. Consistently, an Alphafold model for TsPUF predicted a folded structure with the PUF-like region on the surface of the protein (Fig. 2D). The overall fold of the protein was similar to the Alphafold prediction of *C. elegans* homologue and as these are computational predictions the small differences in orientation of the two major regions of the protein relative to each other may not be represented in the true structures of the molecules (Fig. S2).

**Fig. 3.**
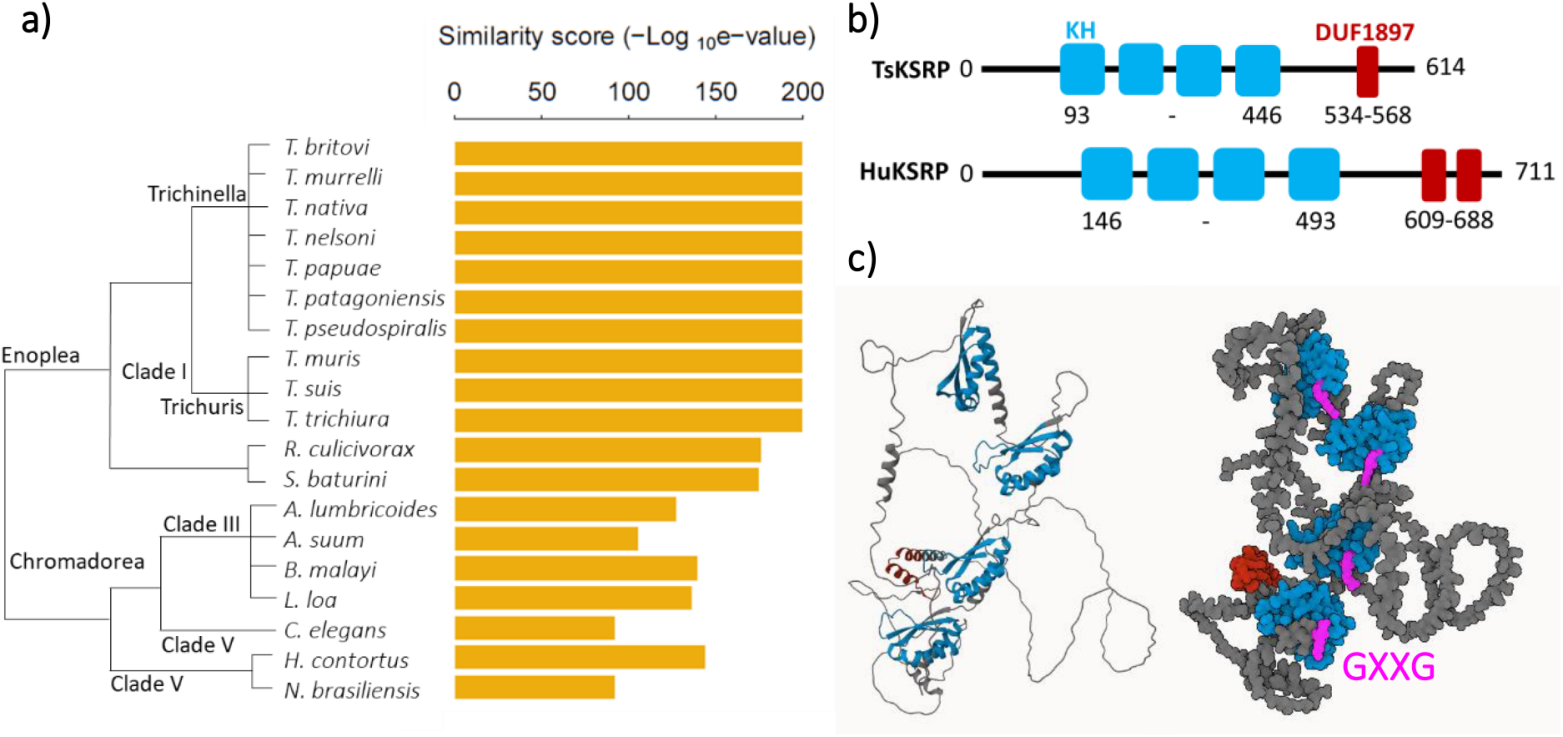
**Characterisation of TsKSRP protein and structure.** (A) Similarity between TsKSRP and its homologues in 19 nematode species; *Trichinella britovi, murrelli, nativa, nelsoni, papuae, patagoniensis* and *pseudospiralis, Trichuris muris, suis* and *trichiura, Romanomermis culicivorax, Soboliphyme baturini, Asacaris lumbricoides* and *suum, Brugia malayi, Loa loa, Caenorhabditis elegans, Haemonchus contortus* and *Nippostrongylus brasiliensis*. Homologues identified by performing a reciprocal best blast hit search. Similarity score=inverse log_10_ of the e value from the reciprocal best blast hit. (B) Comparison of TsKSRP protein domain structure versus that of human KSRP protein (HuKSRP). (C) Alphafold prediction of TsKSRP (A0A0V1B7I9_TRISP) structure. The KH domains are coloured in blue with the GXXG loop in pink. The DUF1897 domain is coloured in dark red.

We next characterised TsKSRP using bioinformatics. TsKSRP was highly conserved across nematodes (Fig. 3A). It was clearly homologous to characterised KSRP from other organisms, showing a similar domain structure to mammalian KSRP (Fig. 3B). No signal peptide was present, nor extracellular domains. Alphafold predicted a folded structure with the GXXG loop, previously implicated in nucleic acid binding (Valverde et al., 2008) exposed to solvent (Fig. 3C). The predicted structure was highly similar to the predicted structure of the *C. elegans* homologue (Fig. S2). This suggested that TsKSRP may have a similar function in intracellular RNA metabolism as in other organisms (Gherzi et al., 2014). In the absence of a canonical signal peptide and with no domains typical of extracellular proteins, TsKSRP may be secreted from cells via alternative routes to the canonical secretory pathway (see Discussion).

### Recombinant TsKSRP and TsPUF bind miRNAs

**Fig. 4.**
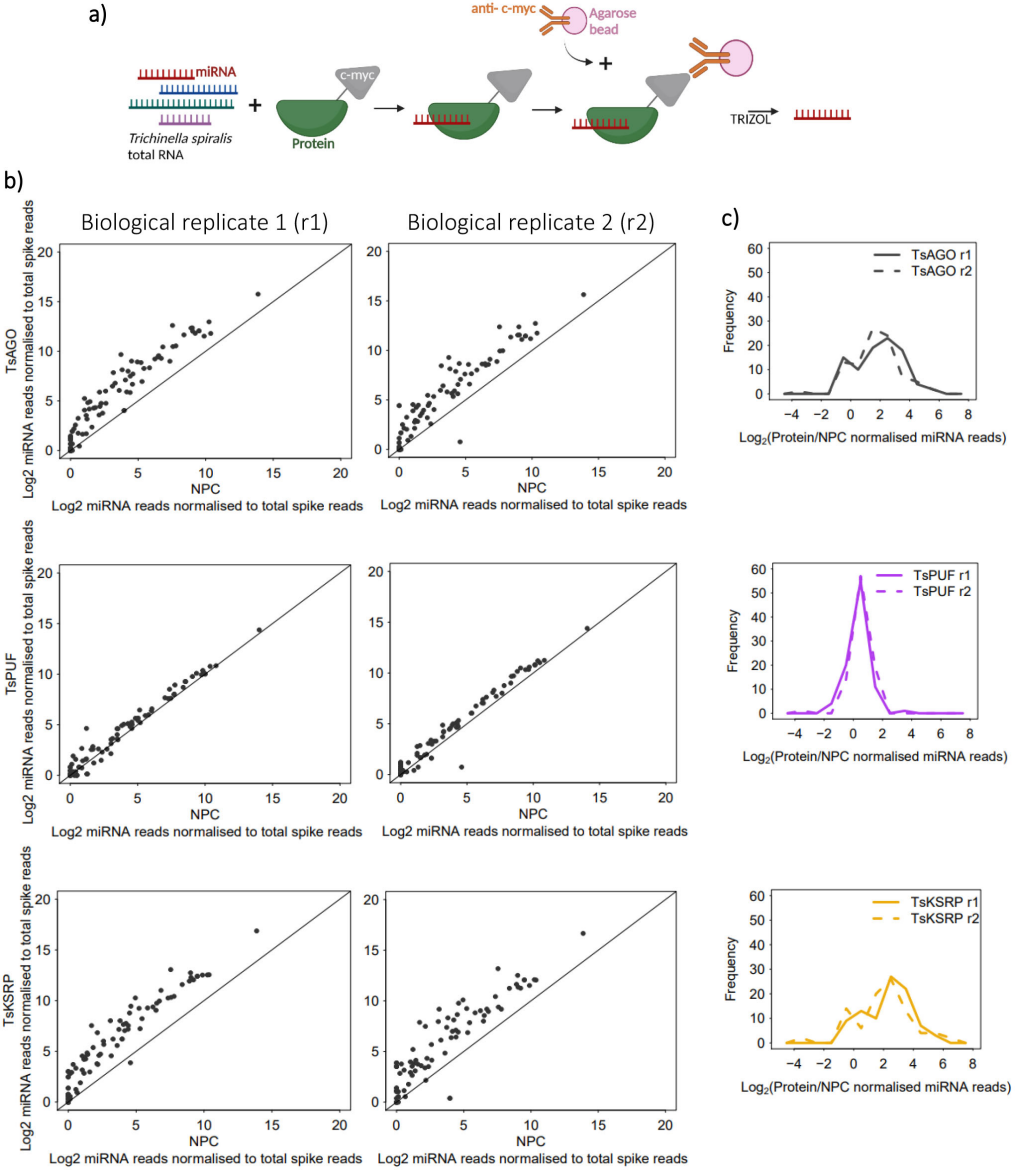
**Analysis of miRNAs pulled-down by *in vitro* RNA immunoprecipitation (RIP) using recombinant TsAGO/PUF/KSRP.** (A) Schematic of RIP assay. (B) Enrichment of *T. spiralis* miRNA reads in two biological replicate (r) RIP reactions containing recombinant proteins (TsAGO/PUF/KSRP), relative to RIP control reactions with no protein (NPC). miRNA reads from sequencing of small RNA libraries were normalised against reads for oligos spiked-in before sRNA library preparation. (C) Distribution of enrichment of *T. spiralis* miRNA reads in RIP reactions containing recombinant TsAGO/PUF/KSRP, relative to a NPC.

To examine whether TsKSRP and TsPUF could contribute to the secretion of miRNAs by *T. spiralis*, we tested whether TsKSRP and TsPUF could bind RNA *in vitro*. We expressed TsPUF with N-terminal his- and c-myc tags in yeast and purified from secreted material using Ni-NTA affinity chromatography. We expressed TsKSRP in bacteria with C-terminal his- and c-myc tags and purified using Ni-NTA affinity. As a positive control, we identified a *T. spiralis* Argonaute homologue (TsAGO) predicted to bind miRNAs (Sarkies et al., 2015), expressed it in bacteria with C-terminal his- and c-myc tags and purified it using Ni-NTA affinity. We incubated all three proteins with total RNA extracted from whole MSL, repurified the proteins using anti-c-myc pulldowns and extracted co-purifying RNA (Fig. 4A). We then subjected co-purifying sRNAs to high-throughput sequencing, adding synthetic short non-coding RNAs with no overlap to the *T. spiralis* genome as normalization controls (see Materials and Methods) (Table S5). The size profile of reads in all reactions is visualised in Fig. S3. We focussed on miRNAs as our aim was to discover the mechanism of miRNA secretion and stability. However, we note that miRNAs make up a small percentage of the reads in all reactions. Around 50% of the remaining small RNAs corresponded to unannotated regions of the genome, with smaller fractions aligning to genes, repetitive elements and structural RNAs such as rRNAs and tRNAs (Fig. S4). The proportions were very similar across the different immunoprecipitated proteins. Future work will be required to investigate the characteristics of non-miRNAs in the secreted material, particularly those that do not align to genomic regions with clear annotations.

TsAGO and TsKSRP both bound selectively, with some miRNAs consistently enriched in the pulldown compared to others (Fig. 4B,C). The distribution of enrichments was bimodal suggesting a small population of highly enriched miRNAs. There was a significant overlap of highly enriched miRNAs in the two biological replicates (Fig. S5). The enrichment of each miRNA was also highly correlated between TsKSRP and TsAGO pulldowns (Fig. S6). Human KSRP has some sequence-specific binding properties, in particular showing preference for G nucleotides in miRNAs (Trabucchi et al., 2009) and discrimination against C nucleotides in all RNA targets (García-Mayoral et al., 2008). We tested whether nucleotide content was different in miRNAs binding to TsKSRP or TsAGO *in vitro*. We found a significant depletion of C nucleotides in miRNAs enriched for TsKSRP binding but no enrichment for G content (Fig. S7). We did not find any significant enrichments for dinucleotides or trinucleotides (Figs S8 and S9).

**Fig. 5.**
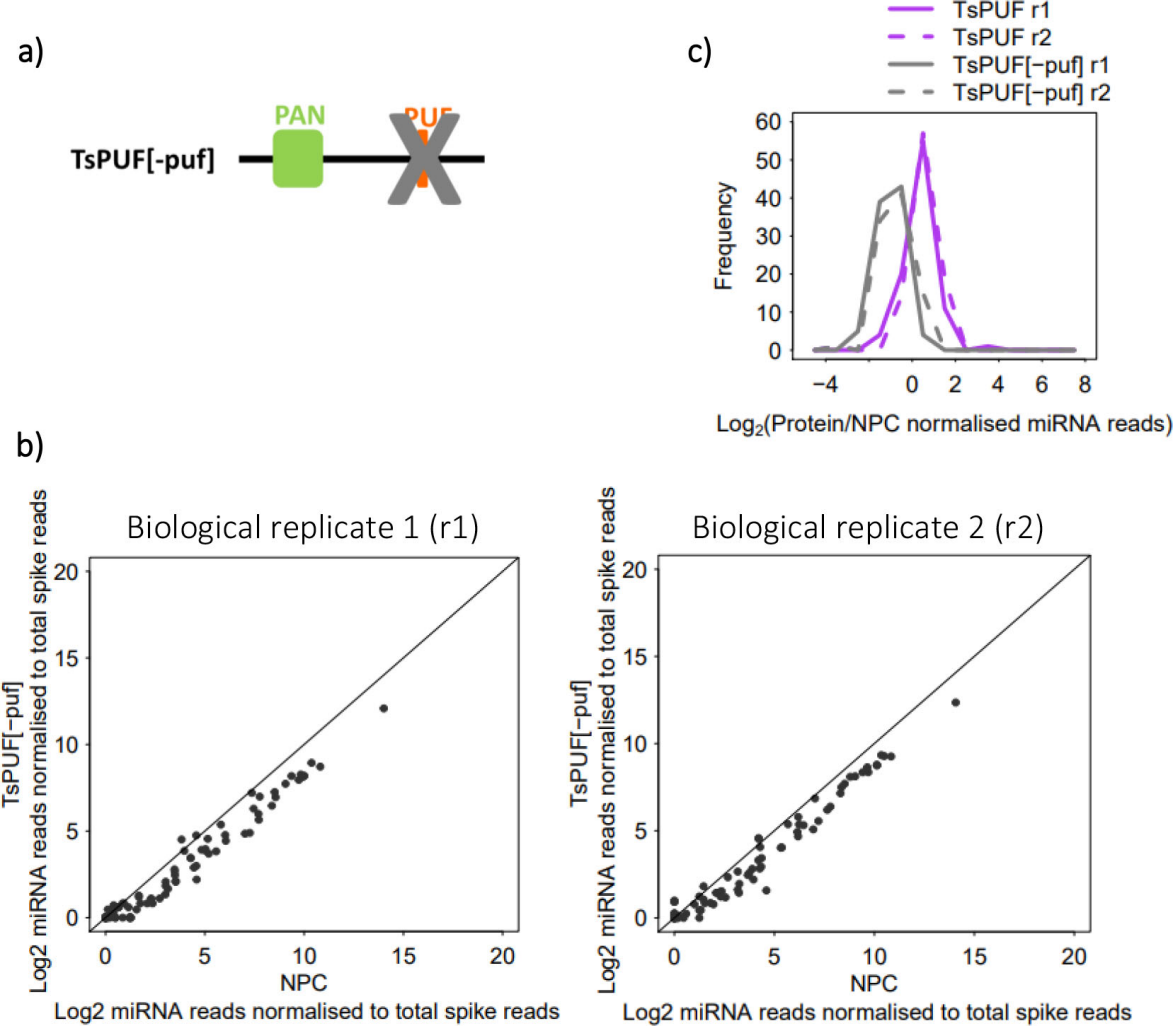
**Analysis of miRNAs pulled-down by *in vitro* RNA immunoprecipitation (RIP) using recombinant TsPUF mutant without PUF domain (TsPUF[-puf]).** (A) Domain structure of TsPUF[-puf] protein. (B) Enrichment of *T. spiralis* miRNA reads in two biological replicate (r) RIP reactions containing TsPUF[-puf], relative to RIP control reactions with no protein (NPC). miRNA reads from sequencing of small RNA libraries were normalised against reads for oligos spiked-in before sRNA library preparation. (C) Distribution of enrichment of *T. spiralis* miRNA reads in RIP reactions containing recombinant TsPUF/TsPUF[-puf], relative to a NPC.

TsPUF exhibited a different pattern of enrichment from TsKSRP and TsAGO, whereby almost all miRNAs bound to a similar extent (Fig. 4B,C). The unimodal distribution of enrichments thus suggested moderate, non-selective binding to most miRNAs (Fig. 4B). Furthermore, the correlations of enrichments between TsPUF and either TsKSRP or TsAGO were weaker than between TsKSRP and TsAGO (Fig. S6; correlation coefficients in Table 1), supporting a different binding mode. We wondered whether the PUF-like region in TsPUF contributed to RNA binding, so we expressed and purified recombinant TsPUF lacking specifically this region (Fig. 5A,B). TsPUF lacking the PUF-like region failed to bind miRNA (Fig. 5C,D). The profile of non-miRNA reads associated was also different in TsPUF lacking the PUF-like region, with a higher proportion coming from ribosomal RNAs (Fig. S4). Thus, despite lack of homology to canonical PUF proteins, the PUF-like region may contribute to RNA binding either directly or through stabilising the correct fold of the protein

**Table 1.**
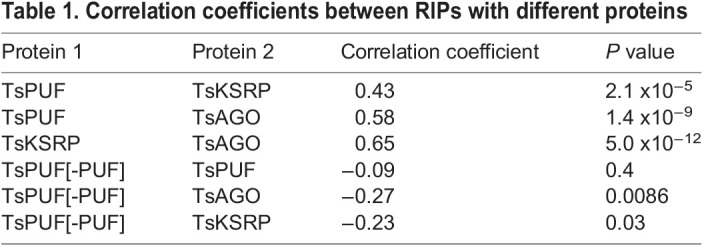
Correlation coefficients between RIPs with different proteins

miRNAs enriched in IPs of TsKSRP, TsAGO and TsPUF all contained some miRNAs that did not have homologues in either *C. elegans*, *H. sapiens* or *D. melanogaster.* Moreover, the *T. spiralis* homologue of the mammalian muscle-specific miRNA miR-31, which we have previously characterised as one of the more abundant miRNAs secreted by MSL, was enriched in IPs with all three proteins (Tables S6,S7 and S8).

Taken together we concluded that the secreted proteins TsKSRP and TsPUF bind miRNAs, but while TsKSRP showed selective binding, similar to the canonical sRNA binding protein TsAGO, TsPUF binds non-selectively to miRNAs.

### TsPUF binds miRNAs in the secreted material from *T. spiralis* larvae

**Fig. 6.**
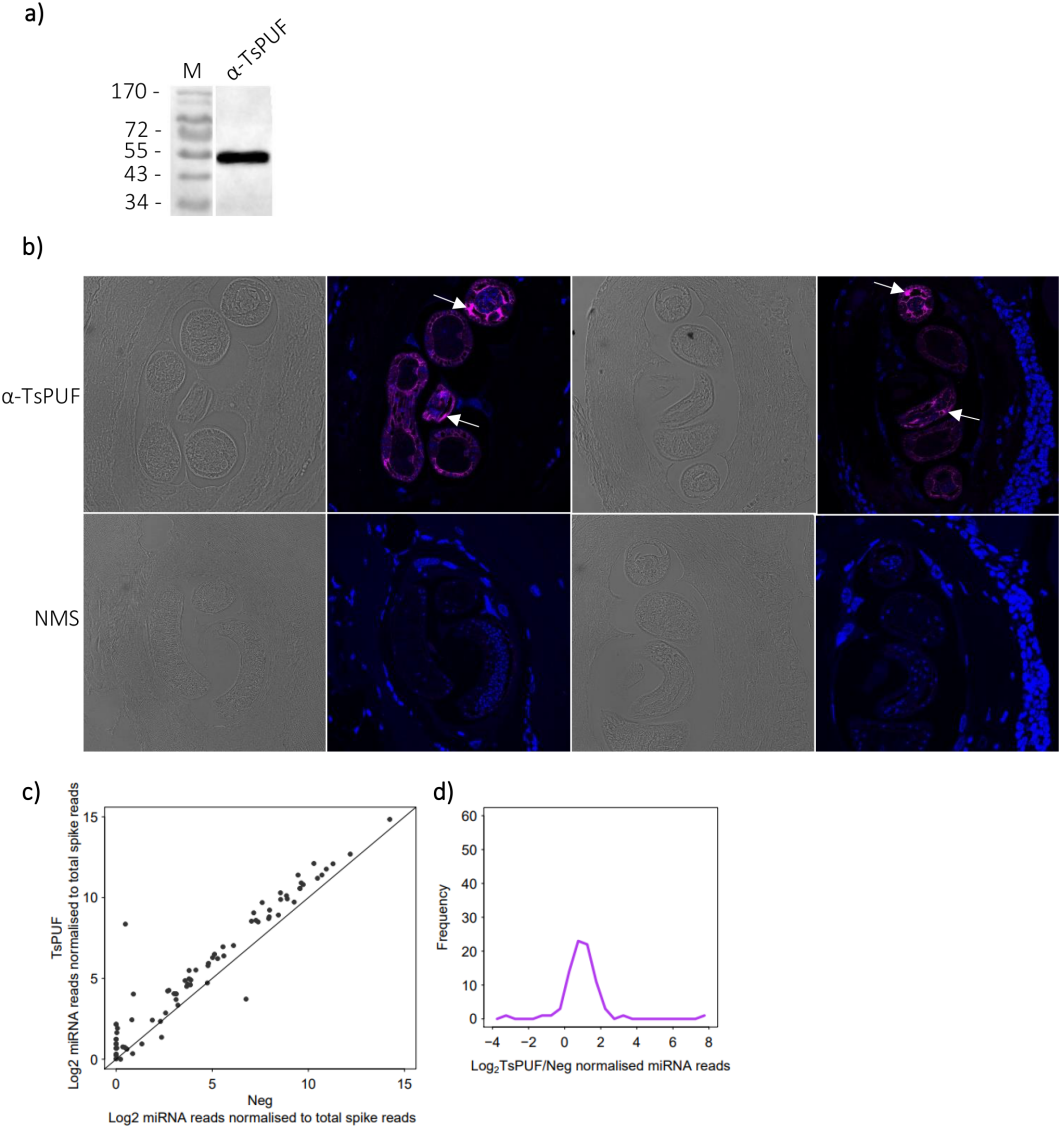
***In vivo* characterisation of native TsPUF in *Trichinella spiralis* muscle-stage larvae (MSL).** (A) Western blot performed on *T. spiralis* MSL secreted material using α-TsPUF antiserum. M=marker, with molecular weight (kDa) indicated. (B) Immunofluorescence staining of *T. spiralis* infected mouse thigh muscle sections. Top panels stained with α-TsPUF antiserum and DAPI counterstain (brightfield and immunofluorescence), with localisation in larval pseudocoelom arrowed. Bottom panels stained with naïve mouse serum (NMS) and DAPI counterstain (brightfield and immunofluorescence). (C) Enrichment of *T. spiralis* miRNA reads by RNA immunoprecipitation (RIP) of native TsPUF, using α-TsPUF antiserum, from MSL secreted material. Enrichment is relative to a RIP control reaction with naïve mouse serum (Neg). miRNA reads from sequencing of small RNA libraries were normalised against reads for oligos spiked-in before sRNA library preparation. (D) Distribution of enrichment of *T. spiralis* miRNA reads by RIP of native TsPUF, relative to Neg.

Having established that recombinant TsKSRP and TsPUF bound to miRNAs *in vitro*, we next wanted to test whether the proteins were present bound to RNA in the secretome of *T. spiralis*. We were not able to raise a specific antibody against TsKSRP. However, we successfully raised an antibody against TsPUF which produced a single, clear band at the correct size when tested by Western blotting on secreted material, indicating specific binding (Fig. 6A). We therefore focussed on TsPUF for this analysis. We visualised the localisation of TsPUF on fixed sections from muscle of infected mice using immunofluorescence. We observed highly specific staining for TsPUF in larvae (Fig. 6B). The protein staining was strongly concentrated in the pseudocoelomic fluid, which bathes the parasite organs. Staining was mutually exclusive with DAPI and cellular structures (Fig. 6B). Together, this was consistent with the prediction that TsPUF is predominantly extracellular, and targeted to the conventional secretory pathway. We did not observe any TsPUF in mouse muscle cytoplasm; however, this does not exclude secretion from the parasite into the host muscle cells, as secreted proteins may be too diffuse in host tissue to be detected.

We next tested whether the protein was bound to miRNAs in secreted material collected from *T. spiralis* MSL in culture. We immunoprecipitated (IP) TsPUF from secreted material and extracted bound RNAs, comparing to IP with naïve mouse serum as a control (Table S9). TsPUF IPs showed clear enrichment of most miRNAs compared to the negative control (Fig. 6C), consistent with relatively non-selective binding. There was a moderate correlation between the enrichment of miRNAs bound to recombinant TsPUF and immunoprecipitated TsPUF (Fig. S10; r=0.50, *P*=3.4×10^−7^). The overall profile of non-miRNA reads was largely similar to those bound *in vitro*, with a slight enrichment of ribosomal RNAs (Fig. S4), and a similar size profile was also seen (Fig. S1^1^). Thus, we concluded that TsPUF binds to miRNAs in the *T. spiralis* MSL secretome non-selectively. Future work to investigate whether TsPUF is bound to other species of RNA in the MSL secretome would be of interest.

## DISCUSSION

Here, using a combination of computational biology, biochemistry and cell biology, we discovered two RNA binding proteins secreted by the parasitic nematode *T. spiralis*. We confirmed that recombinant versions of both proteins bind miRNAs *in vitro*. In the case of one of these proteins, TsPUF, we were able to demonstrate that it bound miRNAs in secreted material from the parasite. Our findings provide new insights into the mechanism whereby miRNAs might be secreted and stabilised in parasitic nematodes and may be relevant for understanding the biology of extracellular RNA more widely.

### Secreted RNA binding proteins in *T. spiralis*

The cellular functions of small non-coding RNAs including miRNAs are largely mediated by proteins of the Argonaute family (AGOs) (Swarts et al., 2014). However, we did not identify any AGO proteins in secreted material from either adult or MSL *T. spiralis*. Nevertheless, we did identify several proteins with domains previously annotated as RNA binding in the secretomes. We characterised two such RNA binding proteins that were enriched in MSL secretory material. One of these proteins, TsKSRP, was a member of a family of known RNA binding proteins. KSRP in other organisms is associated with miRNA sorting and stabilisation (Gherzi et al., 2014), and we showed that TsKSRP has selective binding to some miRNAs. However, it has never been characterised as an extracellular protein. This raises the question of how KSRP is secreted by *T. spiralis*. Importantly, we did not detect a canonical signal peptide in the protein sequence, suggesting that it may not be secreted via the ER secretory pathway. One possibility is that the protein is secreted via exosomes which are prone to lysis, releasing KSRP into extravesicular material.

In contrast, TsPUF contained the PAN domain, often found in extracellular proteins, and had a signal peptide, thus is likely to be secreted via the ER. Identification of this protein as an RNA binding protein was due to a small region that showed weak similarity to a PUF repeat; however, we showed that this is unlikely to reflect homology to the Pumilio family and may be either convergent evolution or a coincidence. Although a deletion encompassing the PUF repeat failed to bind RNA we cannot exclude that this interfered with the overall structure. Interestingly, it showed a very different profile of RNA binding to either TsKSRP or TsAGO, suggesting an entirely different mechanism of RNA binding and stabilisation. Structural characterisation of TsPUF and KSRP would be of great interest in resolving these different binding modes, particularly combined with biophysical measurements of RNA affinity and evaluation of the extent to which it can protect RNA from nuclease activity. Overall, TsPUF is a novel RNA binding protein but the exact mechanism responsible for RNA binding still awaits characterisation. We note that extracellular miRNAs are very common across organisms but there is so far limited evidence for canonical RNA binding proteins involved in their stabilisation (Gruner and McManus, 2021); indeed novel RNA binding proteins in extracellular material have been uncovered (Maori et al., 2019). It is possible that our discovery of TsPUF as an RNA binding protein through domain searches was serendipitous and we predict that there may be many other non-canonical RNA binding proteins with roles in RNA secretion or stabilisation outside cells.

### Insights into the mechanism of RNA secretion by *T. spiralis*

The different properties of KSRP and TsPUF enable us to speculate on the pathway of RNA secretion from *T. spiralis* MSL. KSRP in humans is able to bind to a variety of RNA targets (Palzer et al., 2022), including miRNA precursors via an interaction with the unpaired region of the stem-loop (Trabucchi et al., 2009). This interaction is proposed to underpin the requirement for KSRP in processing specific miRNAs (Trabucchi et al., 2009). KSRP has not been reported as binding to mature miRNAs but given its propensity for single strand RNA binding, it is possible that it could interact with them directly, potentially following Dicer cleavage. Human KSRP shows a preference for binding G-rich miRNAs and selects against cytosine nucleotides (Trabucchi et al., 2009; García-Mayoral et al., 2008). We found that TsKSRP binds selectively to miRNAs and that these miRNAs were depleted of cytosine, although we did not detect enrichment of G within enriched miRNAs. It is therefore possible that TsKSRP aids selective export of miRNAs, using sequence-specific binding. For this to be feasible, folded TsKSRP in complex with miRNA would have to be able to move into the extracellular space. Exactly how this could occur is unclear, but possibilities include a vesicle which lyses after secretion or an as-yet-undiscovered direct route for folded proteins through the plasma membrane (Groot and Lee, 2020). In contrast, TsPUF is most likely to be secreted in an unfolded form through the canonical protein secretion pathway. It would thus only be able to bind to miRNAs once it reaches the extracellular space. We therefore propose that KSRP transfers miRNAs to TsPUF via a hand-off mechanism. As a non-selective binder, TsPUF would therefore acts as a ‘sponge’ to stabilise all extracellular RNAs. Importantly, *T. spiralis* MSL secretes miRNAs that are predominantly vesicle free (Taylor et al., 2020) so TsPUF complexes would have direct access to the host cytoplasm. TsPUF-miRNA complexes may be able to target host genes directly; alternatively, miRNAs could be passed from TsPUF to host Argonaute proteins, which would be a possible route for them to integrate into host gene expression programmes. It will be intriguing to test the extent to which this mechanism operates in muscle cells infected with *T. spiralis* and what role this might play in its pathogenesis. More broadly, we showed that *Trichuris muris*, which also has a partially intracellular lifecycle stage, encodes a homologue of TsPUF with a PUF-like region, so the mechanism we propose may apply to this nematode as well.

## MATERIALS AND METHODS

### Isolation of *T. spiralis* adults and MSL and preparation of total worm extract, secreted material and total worm RNA

Adult parasites were collected from infected rat intestines 6 days post-infection by sedimentation in a Baermann funnel and MSL were recovered from digested mouse muscle 2 months post-infection, as previously described (Arden et al., 1997). For preparation of total worm extracts, adults/MSL were lysed in PBS with 0.05% Tween 20 and protease inhibitors (Sigma-Aldrich, P8340) using a Qiagen TissueLyser II for 20 min at 25 Hz. The lysate was collected as the total worm extract. For preparation of secreted material, parasites were cultured in serum-free medium for up to 72 h as previously described (Arden et al., 1997). Secreted products were collected daily and the supernatants cleared through 0.2 μm filters, pooled and concentrated using 10,000 molecular weight cutoff vivaspin columns. For preparation of total worm RNA, parasites were lysed in TRIZOL using the TissueLyser for 20 min at 25 Hz followed by standard TRIZOL manufacturer's guidelines and RNA precipitation.

### Analysis of *T. spiralis* proteins by mass spectrometry and identification of RNA-binding candidates

Matrix assisted laser desorption ionisation time of flight (MALDI-TOF) mass spectrometry was performed on total worm extracts and secreted material from *T. spiralis* MSL and adults (two technical replicates each). All figures and analysis of the proteomic datasets were performed in RStudio. Protein abundance was defined as the mean normalised intensity value from mass spectrometry. To identify potential RNA-binding proteins in the MSL secretome, all secreted proteins were searched for RNA-binding domains. First, a literature search was performed to create a list of 59 canonical and non-canonical RNA-binding domains (Castello et al., 2016; El-Gebali et al., 2019; Lunde et al., 2007) (Table S2). Next, hmm-scan in the HMMER software (hmmer.org) was used to search the amino acid sequences of all secreted proteins for all domains in the Pfam database (El-Gebali et al., 2019). The identified domains were then cross-matched with the curated list of RNA-binding domains.

### Conservation and bioinformatic characterisation of TsPUF and TsKSRP

The proteomes of *T. spiralis* and other nematode species were downloaded from Wormbase Parasite (Howe et al., 2017) (Table S4). A Blast search (Camacho et al., 2009) was then performed for TsPUF (EFV56078) and TsKSRP (EFV60751) to find hits in each nematode proteome. The reciprocal search was then performed; a Blast search of the hits in each nematode against the *T. spiralis* proteome. The reciprocal best blast hit was defined as a homologue. Clustal Omega (Madeira et al., 2022) was used to perform multiple sequence alignments of the homologues and hmm-scan used to identify Pfam domains. Alphafold (Jumper et al., 2021; Varadi et al., 2022) was used to predict the model structure of TsPUF (A0A0V1BXK5_TRISP) and TsKSRP (A0A0V1B7I9_TRISP). Mol*Viewer (Sehnal et al., 2021) was used to produce model images from the alphafold PDB files.

### Recombinant protein expression in bacteria

Total RNA extracted from *T. spiralis* muscle-stage larvae was reverse transcribed (RT) using Superscript IV reverse transcriptase (ThermoFisher Scientific) following the manufacturer's protocol. A Q5 high fidelity PCR reaction (New England Biolabs) was performed on the cDNA to amplify TsKSRP and TsAGO genes using primers encoding a c-myc tag at the 3′ end. The genes were ligated with a pET21a(+) vector, encoding a his-tag, via compatible restriction digestion sites. Plasmids were replicated and isolated from *Escherichia coli* DH5α for transformation into the expression host *E. coli* BL21. TsKSRP and TsAGO were batch produced via large scale culture of transformed BL21 and induction of protein expression using 1 mM IPTG at 18°C overnight. Cells were lysed with 2.5 mg/ml lysozyme via three freeze-thaw cycles, incubation on ice for 2 h and sonication. Ni-NTA affinity chromatography was performed to purify his-tagged TsAGO and TsKSRP from lysates, following the standard QIAexpressionist protocol.

### Recombinant secretory protein expression in yeast

A TsPUF gene, with sequences encoding a his- and c-myc tag at the N-terminus, was synthesised by GeneArt (ThermoFisher Scientific). The TsPUF mutant without the PUF domain (TsPUF[-puf]) was created by digesting the TsPUF gene with restriction enzymes that cut in positions either side of the sequence encoding the PUF domain and ligating it back together. Both TsPUF[-puf] and the wildtype TsPUF were ligated with a pPICZα vector via compatible restriction digestion sites. Plasmids were replicated and isolated from *E. coli* DH5α for transformation into the expression host *Pichia pastoris*. TsPUF and TsPUF[-puf] were batch produced via large scale culture of transformed *P. pastoris* and induction of protein expression using methanol. Secreted recombinant proteins were collected from the supernatant of the yeast culture. Ni-NTA affinity chromatography was performed to purify his-tagged TsPUF and TsPUF[-puf] from the supernatant, following the standard QIAexpressionist protocol.

### RNA immunoprecipitation using recombinant proteins

Recombinant protein (347 nM) was incubated with total RNA (156 nM) extracted from *T. spiralis* MSL for 30 min in PBS with 0.05% Tween 20 (PBST). The protein:RNA mixture was then incubated with anti- c-myc agarose beads from a Pierce c-Myc Tag IP/Co-IP kit (ThermoFisher Scientific) for 2.5 h. The protein:RNA:anti-c-myc mixture was washed through Pierce spin columns with PBST to remove anything unbound to the anti- c-myc agarose beads. TRIZOL was then used to elute the protein:RNA from the beads. Protein:RNA in TRIZOL was disrupted by repeated freeze-thawing in liquid nitrogen and vigorous vortexing. Chloroform was used to allow phase separation and subsequent precipitation of RNA in the aqueous phase with glycogen and isopropanol. The RNA pellet was washed with 75% EtOH and resuspended in ultrapure water. No-protein-control (NPC) reactions were performed in the exact same way, except with no recombinant protein incubated with the RNA.

### Preparation of cDNA libraries for small RNA sequencing

RNA was used to prepare cDNA libraries with a Truseq sRNA library prep kit (Illumina), following the manufacturer's protocol. Single end sequencing of libraries was kindly performed by the MRC LMS Genomics Laboratory on a NextSeq2000 machine. Oligos were spiked in at the library preparation stage at a constant concentration (0.2% of the input RNA) to allow normalisation of reads from different libraries. To identify appropriate oligos, all human miRNA sequences were downloaded from miRbase (Kozomara et al., 2019) and then a Blast search was performed to identify those with no match against the *T. spiralis* genome. Three sequences were selected in order to have a range of sequence lengths from 19-23 nucleotides long (GGCUUGCAUGGGGGACUGG, UGACAGCGCCCUGCCUGGCUC and GUUUGCACGGGUGGGCCUUGUCU). Oligos were synthesised by Merck.

### Bioinformatic analysis of small RNA sequencing data

Sequencing reads were demultiplexed by the MRC LMS Genomics Laboratory. A shell script was then used to trim adapters and convert to collapsed fasta files with the ‘fastx’ package. To identify *T. spiralis* miRNA, bowtie was used to find the position in the *T. spiralis* genome where the reads align. Bedtools (Quinlan and Hall, 2010) was then used to find reads that align with our previous annotation of miRNAs in the *T. spiralis* genome (Sarkies et al., 2015). All analyses were then performed in RStudio. Information on the first nucleotide and length for each read was extracted using a custom Perl script. For analysis of single nucleotide occurrence in the miRNA sequences, the proportion of each sequence made up of each nucleotide was calculated. The mean nucleotide occurrences were then calculated for miRNA sequences in different enrichment groups. For analysis of di-/tri-nucleotide occurrence in the miRNA sequences, the occurrence of all possible di-/tri-nucleotides were counted in each sequence. These counts were then normalised against the total number of di-/tri-nucleotides present in the given sequence. The mean normalised di-/tri-nucleotide occurrences were then calculated for miRNA sequences in different enrichment groups. To look for significant single/di-/tri-nucleotides, Chi Squared tests, followed by Bonferroni correction, were performed on the mean occurrences of relevant single/di-/tri-nucleotides in different enrichment groups.

### Generation of TsPUF antiserum in a mouse

Endotoxins were removed from purified recombinant TsPUF using Pierce Endotoxin Removal Resin Columns, following the manufacturer's protocol. A mouse was immunised with the endotoxin-free protein mixed with Imject Alum (ThermoFisher Scientific) as an adjuvant. The mouse was then boosted with more protein/adjuvant mix 4 weeks later, and twice more 2 weeks apart before bleeding the mouse 1 week after the final boost. The supernatant was then collected from the blood and this was used as the TsPUF antiserum.

### Immunofluorescence on infected muscle tissue to visualise TsPUF

A section of thigh muscle from an infected mouse was fixed in 10% neutral buffered formalin, embedded in paraffin and sectioned using standard techniques. The sections were deparaffinized and rehydrated via a series of washes with xylene and decreasing concentrations of ethanol. The sections were blocked with 1% bovine serum albumin (BSA) in PBS with 0.1% Tween 20 (PBST) and 10% naïve goat serum. A second block was performed (for mouse-on-mouse staining) using goat F(ab) anti-mouse IgG (Abcam) at 1:100 in PBST. Sections were then incubated with TsPUF antiserum (or naïve mouse serum) at 1:200 in PBST with 1% BSA. Goat anti-mouse alexa fluor 488 (Abcam) at 1:500 in PBST with 1% BSA was then used to stain the sections. The sections were then counterstained using mounting medium containing DAPI (Abcam). Immunofluorescence staining was visualised using a Leica SP8 - STELLARIS 5 Inverted Light Sheet Confocal Microscope. Localisation was determined by reference to the structure of infective larvae (Takahashi, 2021).

### RNA immunoprecipitation of native TsPUF from MSL secreted material

Immunoprecipitations were performed with 10 ul TsPUF antiserum (or 10 ul naïve mouse serum), incubated with 100 ug (protein concentration) *T. spiralis* MSL secreted material in PBS with 0.05% Tween 20 (PBST) for 1 h. The antiserum:secreted material mixture was then incubated with 50 ul Dynabeads Protein G magnetic beads (ThermoFisher Scientific) for 30 min. A magnetic plate was used for washing with PBST. TRIZOL was then used to elute the protein:RNA from the beads. RNA was then isolated following the same method used in the recombinant protein RNA immunoprecipitations.

## Supplementary Material

10.1242/biolopen.060096_sup1 Supplementary information

Table S1. Raw and processed data for the intensity level of all proteins identified by mass spectrometry in *Trichinella spiralis* muscle-stage larvae (MSL) and adult secreted material and total worm extracts.

Table S2. List of RNA-binding domains (RBDs) used in this study. List created by performing a literature search for canonical/non-canonical RBDs.

Table S3. Candidate RNA-binding domain-containing proteins which were more enriched in the secreted material of *Trichinella spiralis* muscle-stage larvae (MSL) than by the secreted material of adults. Abundance of all proteins is also shown (mean normalised intensity, from mass spectrometry, of two replicates).

Table S4. Nematodes used in this study to analyse the conservation of *Trichinella spiralis* proteins.

Table S5. *Trichinella spiralis* miRNA read counts, normalised to total oligo spike counts, from small RNA sequencing of RNA immunoprecipitated using recombinant TsKSRP, TsAGO, TsPUF and TsPUF[-puf]. Data for no protein controls (NPC) are also shown. Two biological replicates performed.

Table S6. Homologues of *Trichinella spiralis* miRNAs enriched by immunoprecipitation of recombinant TsPUF, relative to a no protein control (NPC). Only those miRNAs where log_2_(TsPUF/NPC) > 0 in two biological replicates were defined as enriched.

Table S7. Homologues of *Trichinella spiralis* miRNAs enriched by immunoprecipitation of recombinant TsKSRP, relative to a no protein control (NPC). Only those miRNAs where log_2_(TsKSRP/NPC) > 1 in two biological replicates were defined as enriched.

Table S8. Homologues of *Trichinella spiralis* miRNAs enriched by immunoprecipitation of recombinant TsAGO, relative to a no protein control (NPC). Only those miRNAs where log_2_(TsAGO/NPC) > 1 in two biological replicates were defined as enriched.

Table S9. *Trichinella spiralis* miRNA reads, normalised to total oligo spike counts, from small RNA sequencing of RNA immunoprecipitated from *T. spiralis* secreted material in a native TsPUF pull-down. Data for a negative control (Neg) is also shown.

Table S10. Homologues of *Trichinella spiralis* miRNAs enriched by immunoprecipitation of native TsPUF, relative to a negative control. Only those miRNAs where log_2_(TsPUF/Neg) > 0 were defined as enriched.
