# Supplementary material for: Identification of proteins that bind extracellular microRNAs secreted by the parasitic nematode Trichinella spiralis
Source: Biol Open. 2023 Nov 9;12(11):bio060096. doi: 10.1242/bio.060096 (PMC10660789; doi:10.1242/bio.060096)
Supplement: Supplementary information [file biolopen-12-060096-s1.pdf]

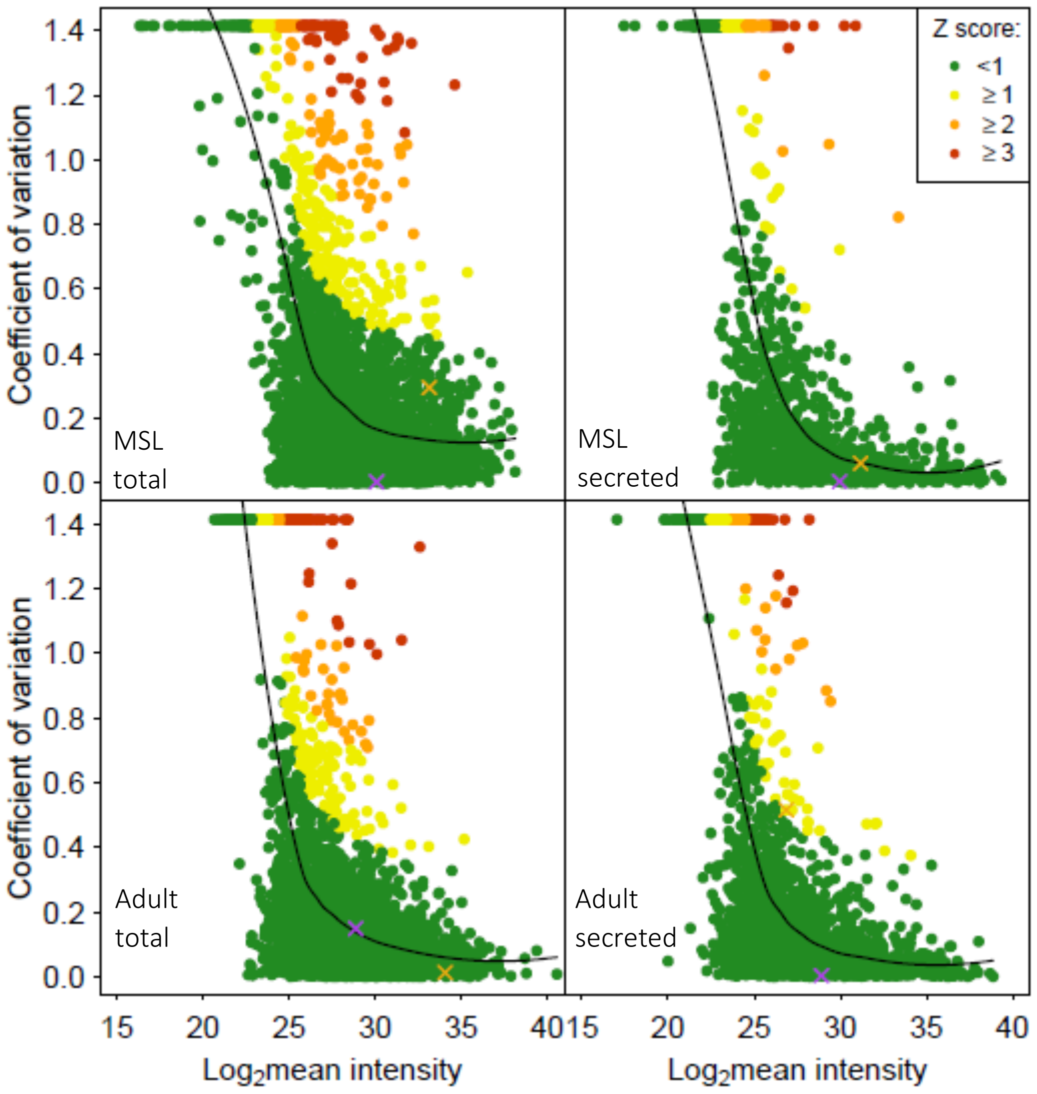

Fig. S1. Intensity value variation between two replicates of all individual proteins identified in mass spectrometry dataset. A best fit curve (black) of the coefficient of variation (CV) against the log2(mean intensity) for each protein was predicted using the loess() function in R. Subsequently calculated residual Z scores of the CVs from the best fit are highlighted. MSL = muscle-stage larvae. The position of TsPUF and TsKSRP are highlighted (X) in purple and orange, respectively.

Biology Open • Supplementary information

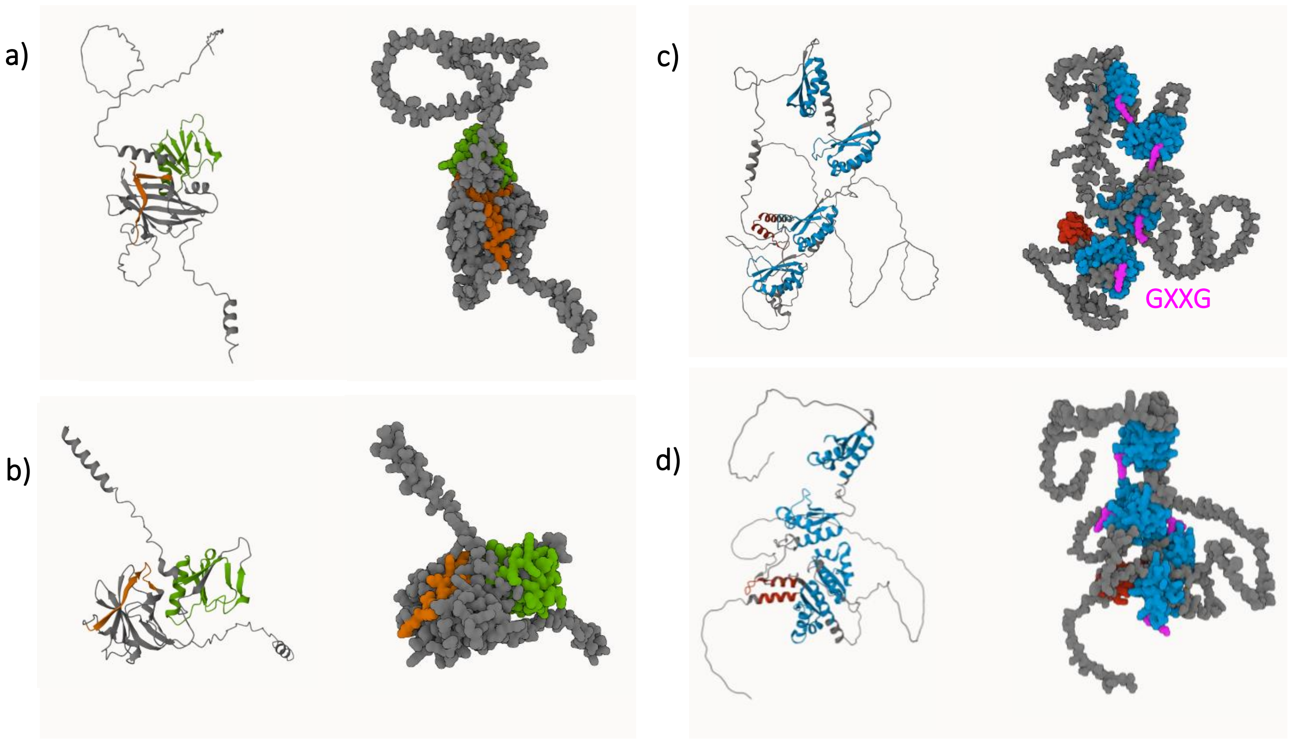

Fig. S2. Alphafold prediction of the structure of *Trichinella spiralis* proteins compared to that of their homologues in *Caenorhabditis elegans*. **(a)** *T. spiralis* TsPUF (A0A0V1BXK5_TRISP). The PUF domain is coloured in orange and the PAN domain in green. **(b)** *C. elegans* TsPUF homologue (Q09271). The region that aligns with the PUF domain in TsPUF is coloured in orange. **(c)** *T. spiralis* TsKSRP (A0A0V1B7I9_TRISP). The KH domains are coloured in blue with the GXXG loop in pink. The DUF1897 domain is coloured in dark red. **(d)** *C. elegans* TsKSRP homologue (Q23487).

Biology Open • Supplementary information

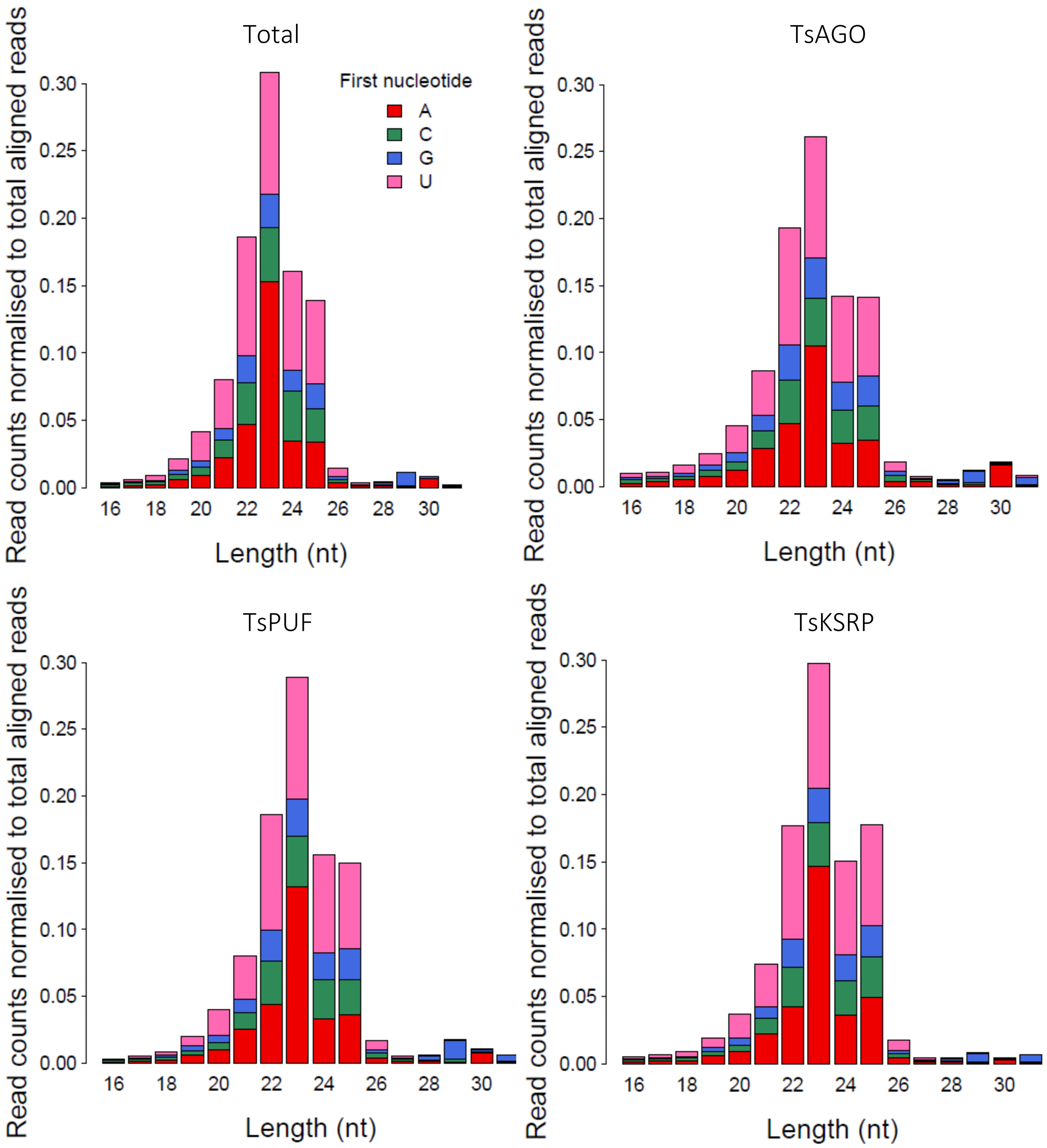

Fig. S3. Profile of sequencing reads, in terms of length (in nucleotides; nt) and nt in the first position, of reads from *in vitro* RNA immunoprecipitation (RIP) reactions. RIP read profiles represent the mean of two biological replicates. Profile of reads from sequencing of total RNA extracted from *Trichinella spiralis* muscle-stage larvae is also shown (Total).

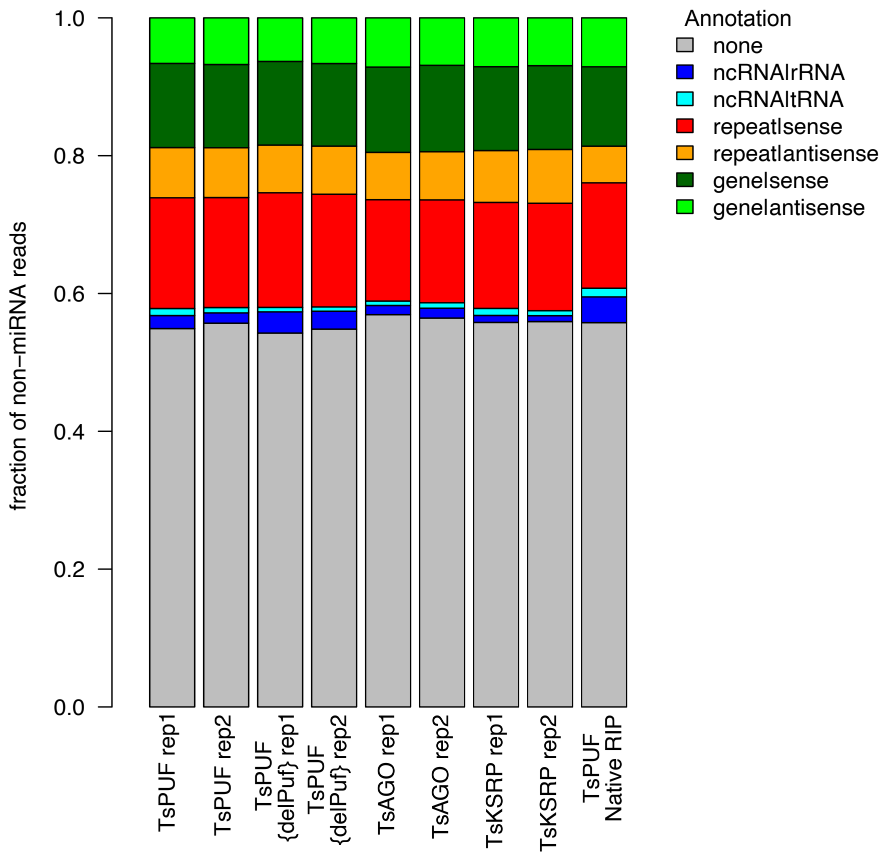

Fig. S4. Alignment Profile of non-miRNA reads in in vitro IP reactions. All reads that aligned to the genome but did not overlap with miRNA loci are shown divided according to the annotation of the overlapping region of the alignment.

Biology Open • Supplementary information

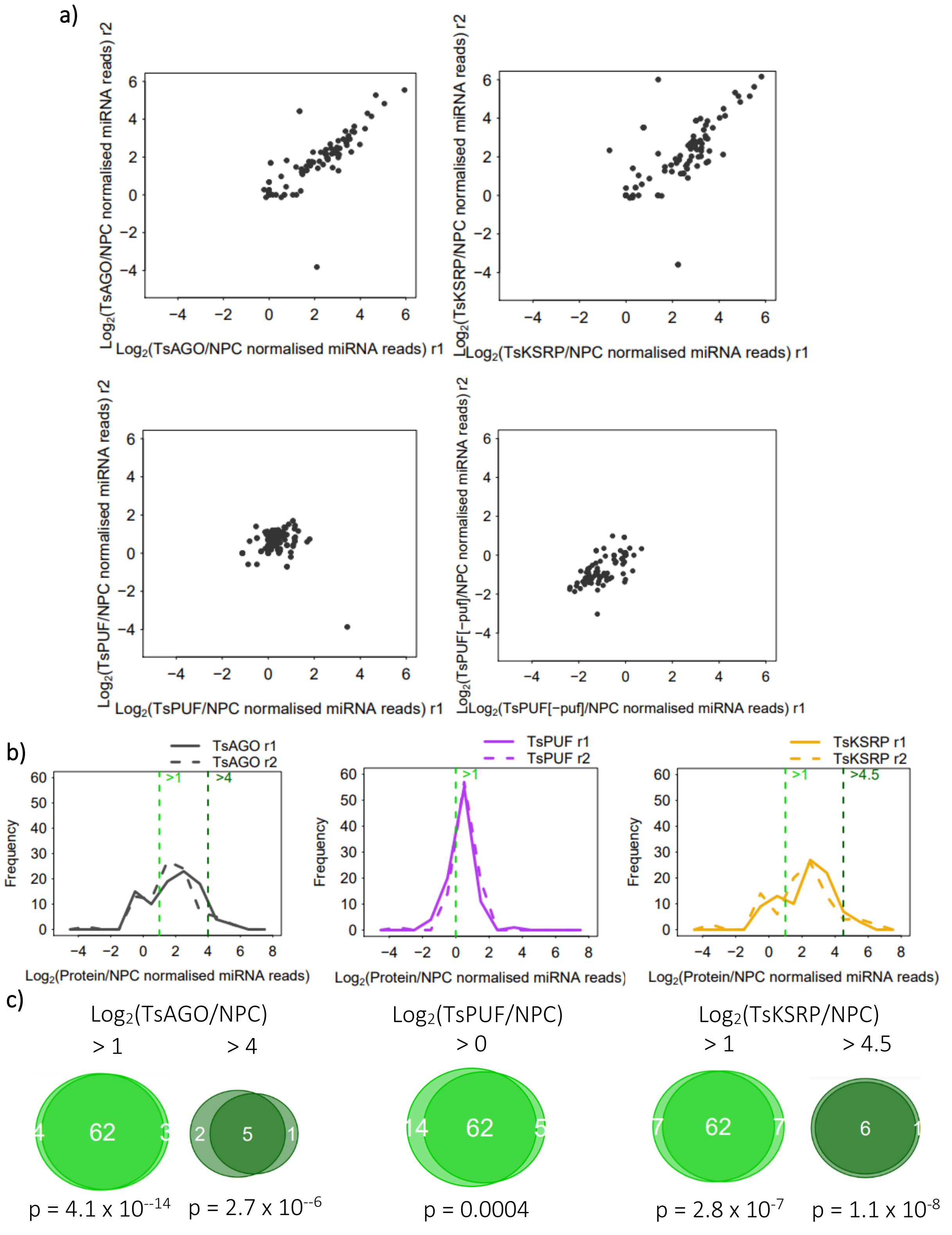

Fig. S5. Comparison of miRNAs pulled down by *in vitro* RNA immunoprecipitation (RIP) in two biological replicates (r). **(a)** Enrichment of *T. spiralis* miRNA reads in protein RIPs, relative to no protein control (NPC) RIPs, in r1 versus r2. **(b)** Distribution of enrichment of *T. spiralis* miRNA reads in protein RIP reactions. Thresholds to define the level of enrichment (in terms of log_2(Protein/NPC)) are labelled in green and dark green. **(c)** Venn diagrams to show the overlap between miRNAs enriched in the two replicates. P values from Fisher's exact test of independence.

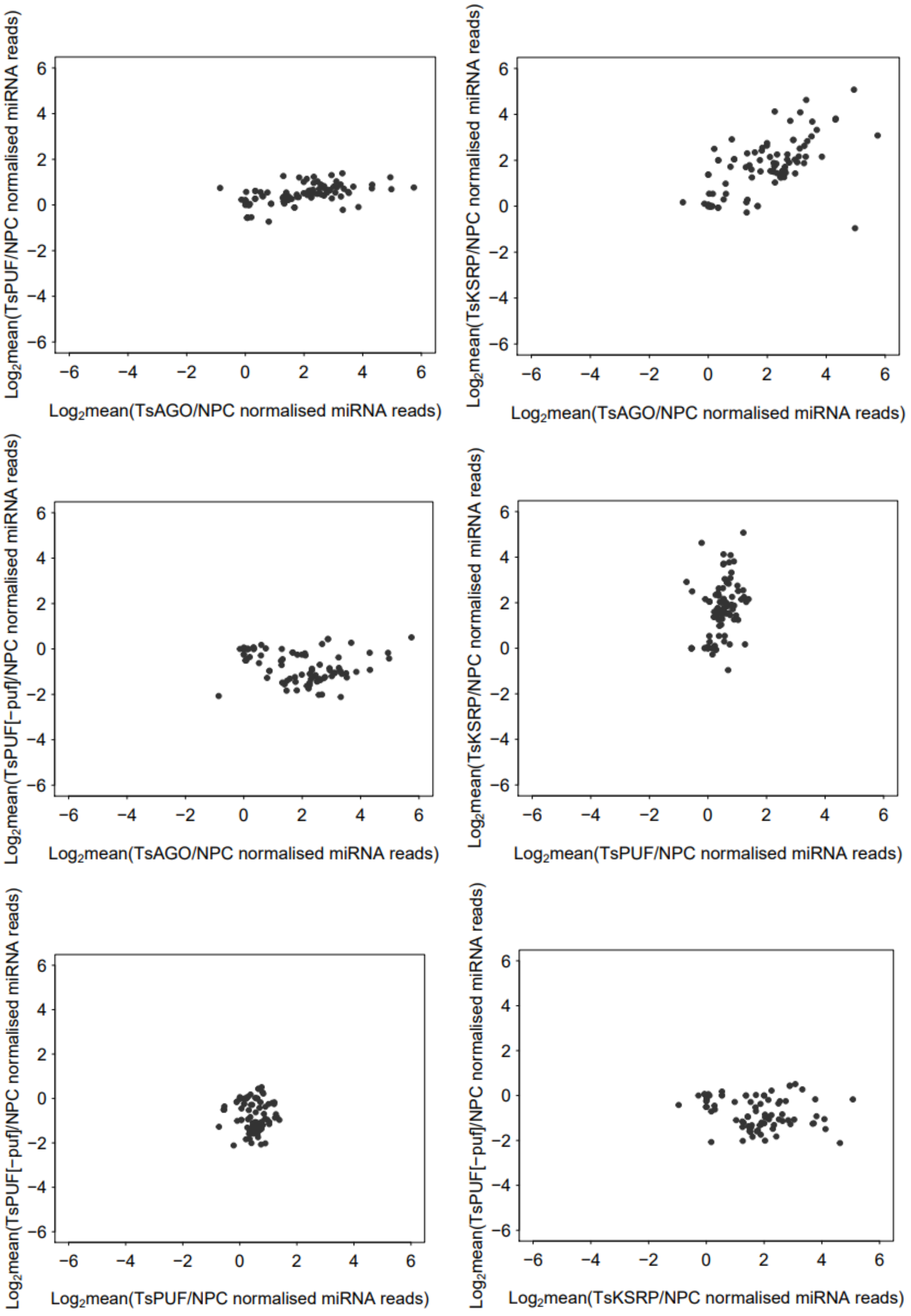

Fig. S6. Comparison of the level of enrichment, relative to no protein control (NPC), of miRNAs by *in vitro* RNA immunoprecipitation (RIP) with different recombinant proteins. Enrichment values represent the mean of two biological replicate RIP reactions.

Biology Open • Supplementary information

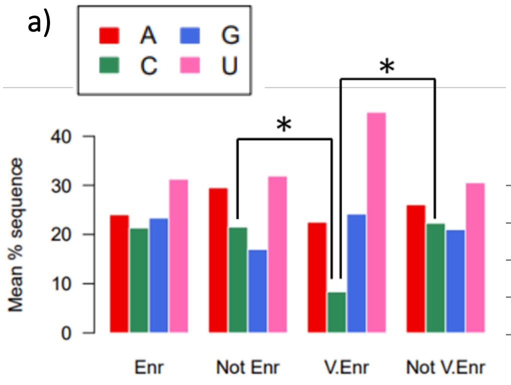

Definitions:

Enriched (Enr)	log2(TsKSRP/NPC) > 1
Not enriched (Not Enr)	log2(TsKSRP/NPC) < 1
Very enriched (V.Enr)	log2(TsKSRP/NPC) > 4.5
Not very enriched (Not V.Enr)	log2(TsKSRP/NPC) < 4.5

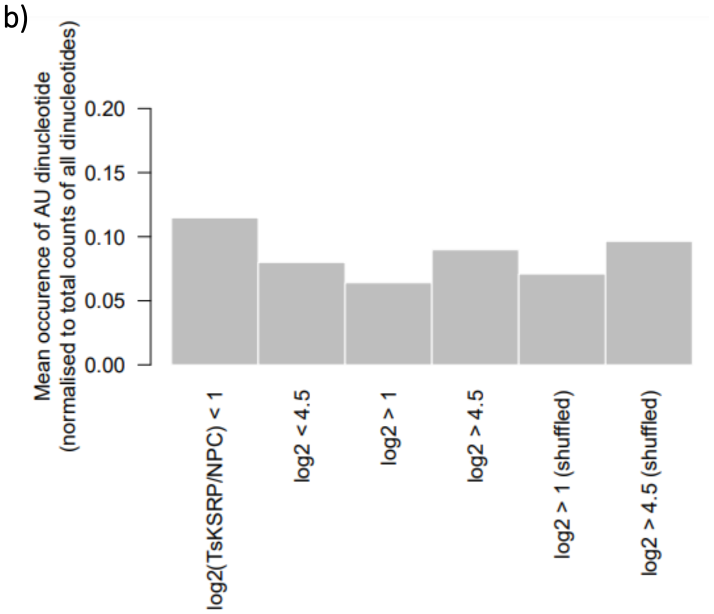

Fig. S7. Global nucleotide **(a)** and AU dinucleotide **(b)** abundance in miRNAs (very) enriched, versus not (very) enriched, by recombinant TsKSRP in RNA immunoprecipitation (RIP) reactions. Definitions of miRNA enrichment, relative to a RIP control reaction with no protein (NPC), are shown. **(a)** For every enrichment group, the proportion (%) of each sequence made up of each nucleotide was calculated. Values shown here are the average (mean) of these proportions within the group. Chi Squared tests, followed by Bonferroni correction, were performed on the mean values for every nucleotide to compare the following groups: Enr versus Not Enr, V.Enr versus Not Enr, V.Enr versus Not V.Enr. **(b)** For every enrichment group, the occurrence of AU in each sequence was counted and normalised against the total number of all dinucleotides in that sequence. Values shown are the mean normalised occurrences. Chi Squared tests were performed on the mean values to compare the following groups: enriched versus not enriched, enriched versus very enriched, enriched versus enriched shuffled, very enriched versus not enriched, very enriched versus not very enriched and very enriched versus very enriched shuffled. Only the differences with significant (<0.1) p values are labelled (*).

Biology Open • Supplementary information

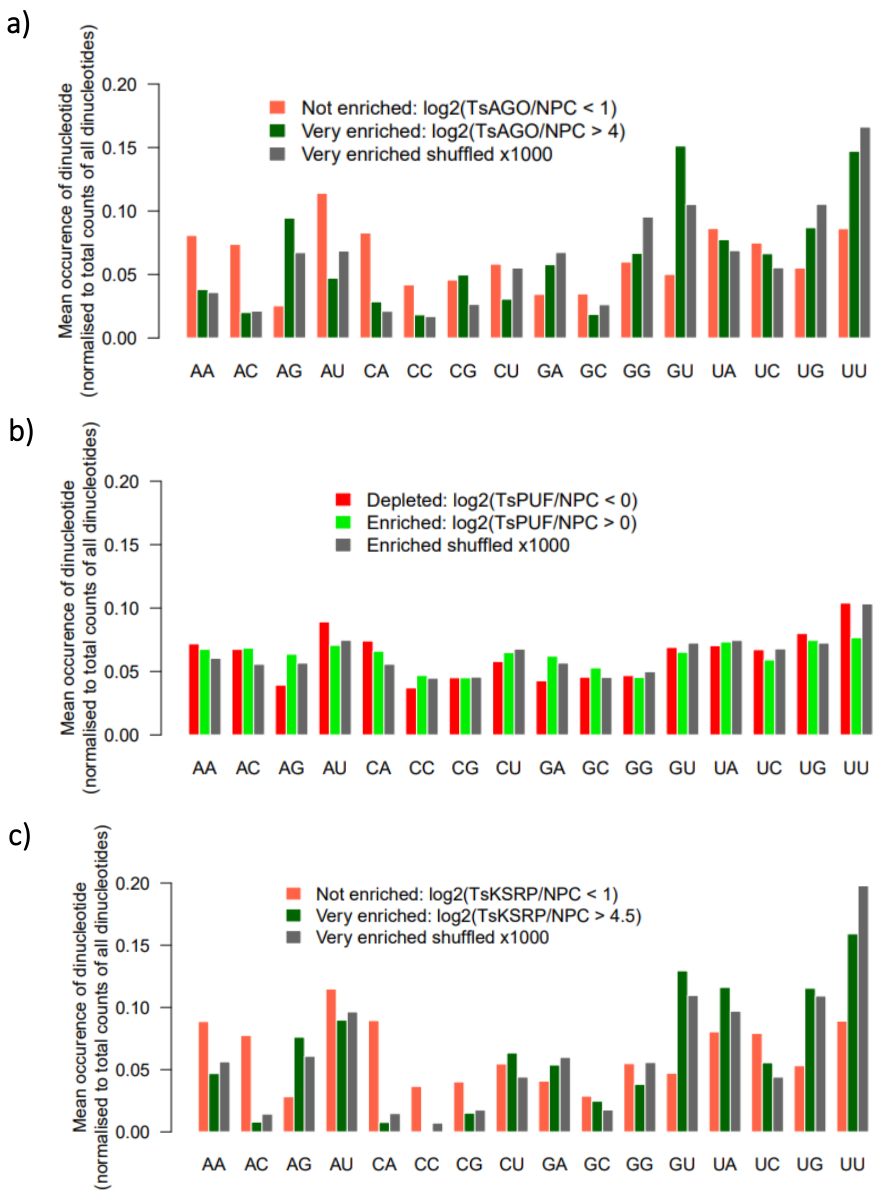

Fig. S8. Global dinucleotide abundance in miRNAs (very) enriched, versus not enriched/depleted, by recombinant TsAGO**(a)**/TsPUF**(b)**/TsKSRP**(c)** in RNA immunoprecipitation (RIP) reactions. For every enrichment group, the occurrence of each dinucleotide in each sequence was counted and normalised against the total number of all dinucleotides in that sequence. Values shown are the mean normalised occurrences. Chi Squared tests, and Bonferroni correction, were performed on the mean values for every dinucleotide to compare the following groups: very enriched versus not enriched and very enriched versus very enriched sequences shuffled x1000 (for TsAGO/TsKSRP). For TsPUF, enriched versus depleted and enriched versus enriched sequences shuffled x1000 were compared. No significant differences were found in any group.

Biology Open • Supplementary information

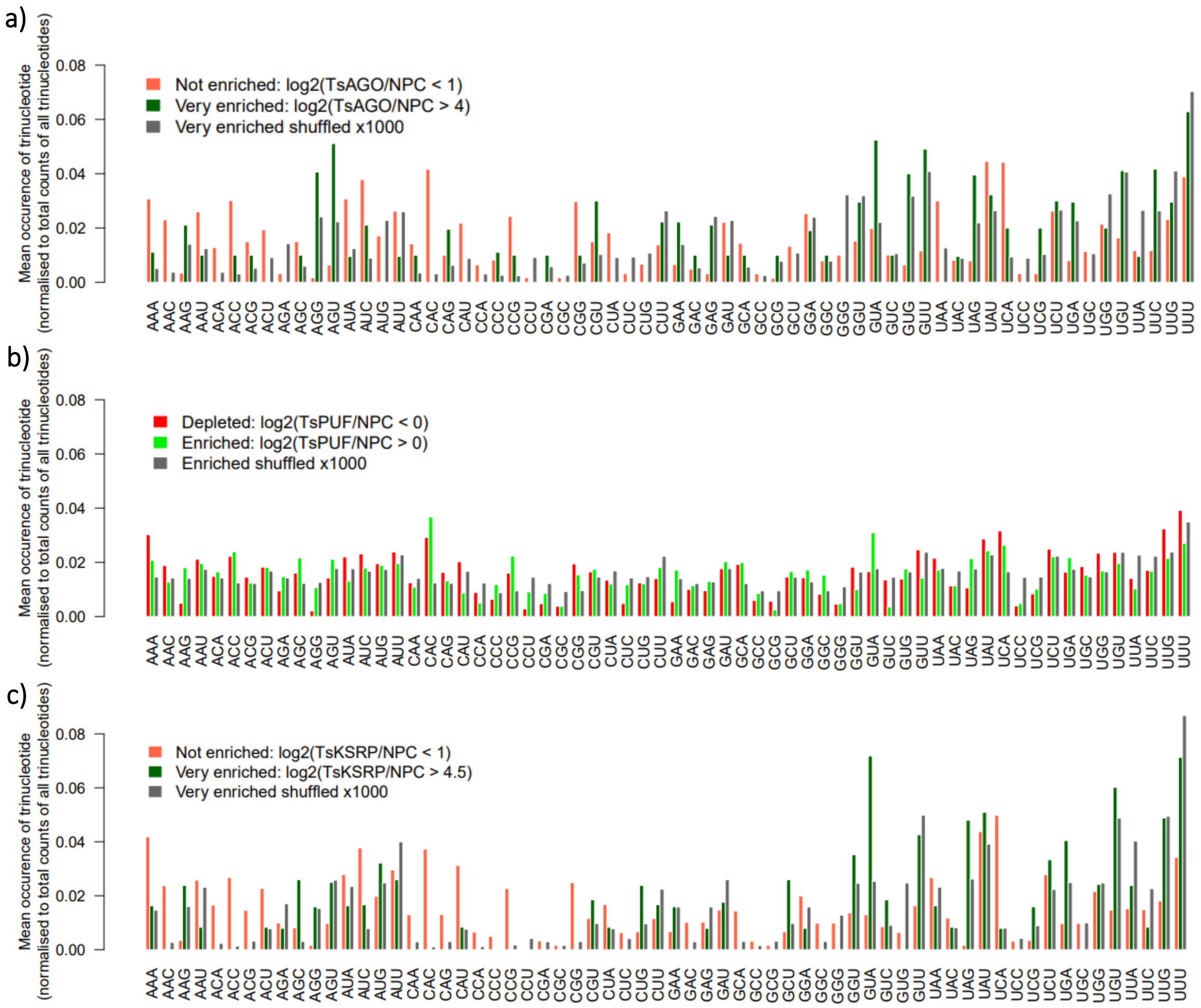

Fig. S9. Global trinucleotide abundance in miRNAs (very) enriched, versus not enriched/ depleted, by recombinant TsAGO**(a)**/TsPUF**(b)**/TsKSRP**(c)** in RNA immunoprecipitation (RIP) reactions. For every enrichment group, the occurrence of each trinucleotide in each sequence was counted and normalised against the total number of all trinucleotides in that sequence. Values shown are the mean normalised occurrences. Chi Squared tests, and Bonferroni correction, were performed on the mean values for every trinucleotide to compare the following groups: very enriched versus not enriched and very enriched versus very enriched sequences shuffled x1000 (for TsAGO/ TsKSRP). For TsPUF, enriched versus depleted and enriched versus enriched sequences shuffled x1000 were compared. No significant differences were found in any group.

Biology Open • Supplementary information

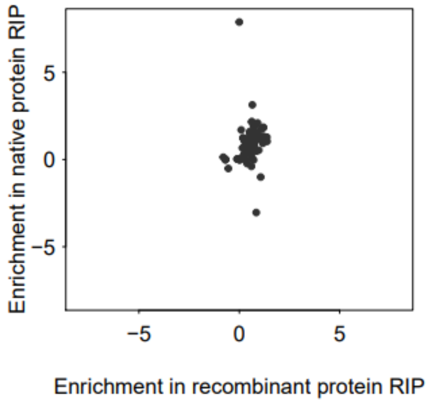

Fig. S10. Comparison of the level of enrichment of miRNAs by *in vitro* RNA immunoprecipitation (RIP) using recombinant TsPUF versus the level of enrichment by RIP of native TsPUF. Enrichment values are relative to a negative control (log_2(protein/negative normalised miRNA reads)). The negative control is either a RIP reaction with no protein (for recombinant protein RIPs) or a RIP using naïve mouse serum (for native RIPs with anti-serum against the native proteins). Enrichment values for the recombinant RIP represents the mean of two biological replicate RIP reactions.

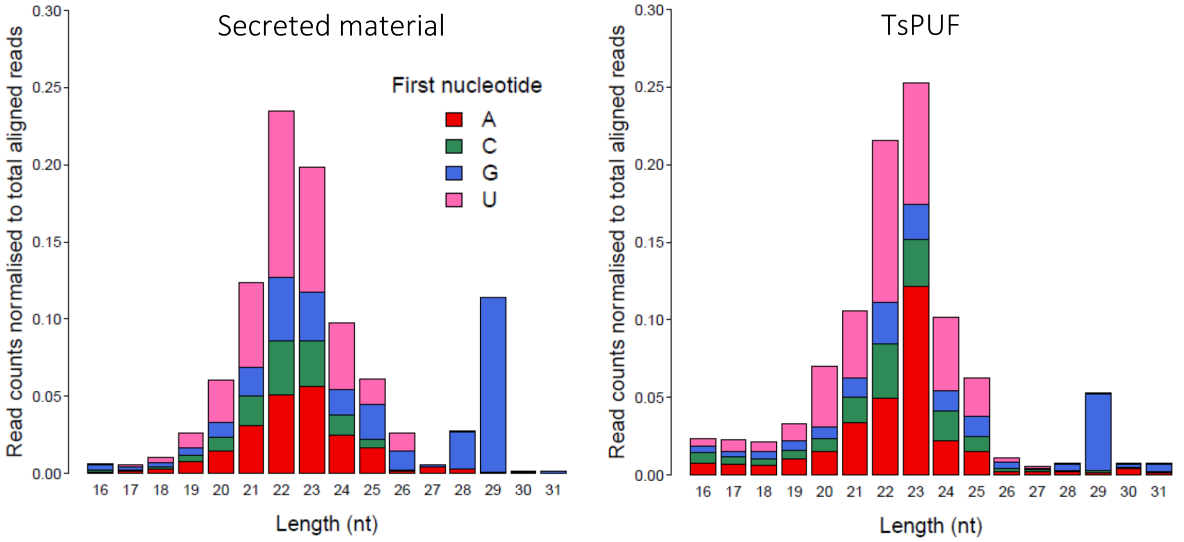

Fig. S11. Profile of sequencing reads, in terms of length (in nucleotides; nt) and nt in the first position, of reads from RNA immunoprecipitation (RIP) of native TsPUF from *Trichinella spiralis* muscle-stage larvae (MSL) secreted material. Profile of reads from sequencing of RNA extracted from *T. spiralis* MSL secreted material is also shown.

Biology Open • Supplementary information

Table S1. Raw and processed data for the intensity level of all proteins identified by mass spectrometry in *Trichinella spiralis* muscle-stage larvae (MSL) and adult secreted material and total worm extracts.

Available for download at
https://journals.biologists.com/bio/article-lookup/doi/10.1242/bio.060096#supplementary-data

Table S2. List of RNA-binding domains (RBDs) used in this study. List created by performing a literature search for canonical/non-canonical RBDs.

Available for download at
https://journals.biologists.com/bio/article-lookup/doi/10.1242/bio.060096#supplementary-data

Table S3. Candidate RNA-binding domain-containing proteins which were more enriched in the secreted material of *Trichinella spiralis* muscle-stage larvae (MSL) than by the secreted material of adults. Abundance of all proteins is also shown (mean normalised intensity, from mass spectrometry, of two replicates).

Available for download at
https://journals.biologists.com/bio/article-lookup/doi/10.1242/bio.060096#supplementary-data

Biology Open • Supplementary information

Table S4. Nematodes used in this study to analyse the conservation of *Trichinella spiralis* proteins.

Available for download at
https://journals.biologists.com/bio/article-lookup/doi/10.1242/bio.060096#supplementary-data

Table S5. *Trichinella spiralis* miRNA read counts, normalised to total oligo spike counts, from small RNA sequencing of RNA immunoprecipitated using recombinant TsKSRP, TsAGO, TsPUF and TsPUF[-puf]. Data for no protein controls (NPC) are also shown. Two biological replicates performed.

Available for download at
https://journals.biologists.com/bio/article-lookup/doi/10.1242/bio.060096#supplementary-data

Table S6. Homologues of *Trichinella spiralis* miRNAs enriched by immunoprecipitation of recombinant TsPUF, relative to a no protein control (NPC). Only those miRNAs where $\log_2$(TsPUF/NPC) > 0 in two biological replicates were defined as enriched.

Available for download at
https://journals.biologists.com/bio/article-lookup/doi/10.1242/bio.060096#supplementary-data

Table S7. Homologues of *Trichinella spiralis* miRNAs enriched by immunoprecipitation of recombinant TsKSRP, relative to a no protein control (NPC). Only those miRNAs where $\log_2$(TsKSRP/NPC) > 1 in two biological replicates were defined as enriched.

Available for download at
https://journals.biologists.com/bio/article-lookup/doi/10.1242/bio.060096#supplementary-data

Biology Open • Supplementary information

Table S8. Homologues of *Trichinella spiralis* miRNAs enriched by immunoprecipitation of recombinant TsAGO, relative to a no protein control (NPC). Only those miRNAs where $\log_2$(TsAGO/NPC) > 1 in two biological replicates were defined as enriched.

Available for download at
https://journals.biologists.com/bio/article-lookup/doi/10.1242/bio.060096#supplementary-data

Table S9. *Trichinella spiralis* miRNA reads, normalised to total oligo spike counts, from small RNA sequencing of RNA immunoprecipitated from *T. spiralis* secreted material in a native TsPUF pull-down. Data for a negative control (Neg) is also shown.

Available for download at
https://journals.biologists.com/bio/article-lookup/doi/10.1242/bio.060096#supplementary-data

Table S10. Homologues of *Trichinella spiralis* miRNAs enriched by immunoprecipitation of native TsPUF, relative to a negative control. Only those miRNAs where $\log_2$(TsPUF/Neg) > 0 were defined as enriched.

Available for download at
https://journals.biologists.com/bio/article-lookup/doi/10.1242/bio.060096#supplementary-data
